# A Comparative Analysis on the Variation of β‐Carotene, Vitamin C and E Levels in Hydroponic and Soil‐Based Fruits and Vegetables in Kiambu County, Kenya

**DOI:** 10.1002/fsn3.70403

**Published:** 2025-06-05

**Authors:** Rhodah Nekesa, Lucy G. Njue, George O. Abong

**Affiliations:** ^1^ Department of Food Science Nutrition and Technology University of Nairobi Nairobi Kenya

**Keywords:** antioxidants, fruits and vegetables, hydroponic system, nutrient accumulation, soil‐based system

## Abstract

Antioxidants such as β‐carotene, vitamin C, and E in fruits and vegetables (FAVs) have been associated with a reduction in non‐communicable diseases because of their vital metabolic roles. However, their levels in foods could vary depending on the farming systems. Hence, this study examined the levels of β‐carotene, vitamin C, and E in hydroponic and soil‐grown spinach, tomatoes, and strawberries. A cross‐sectional study with an analytical component was conducted to facilitate this comparison. Ten samples were collected from hydroponic farms and nine were from soil‐based farms. The data was analyzed using SPSS (version 25). The level of vitamin C was significantly higher in hydroponically grown tomatoes (*p* = 0.008) and strawberries (*p* = 0.037) compared to those from the soil‐based system. The level of vitamin E was significantly higher in hydroponic tomatoes, spinach, and strawberries (*p* ≤ 0.05) compared to those grown in the soil‐based system. Β‐carotene level was significantly higher only in the hydroponically grown spinach (*p* < 0.05) unlike tomatoes, and it was not detected in both hydroponic and soil‐based strawberries. In conclusion, the findings of this study show a remarkable nutrient accumulation in some FAVs grown in the hydroponic farming system leading to a higher nutrient density compared to those grown in the soil‐based systems. Training and provision of resources for hydroponic farming could be done to scale up production in the country to promote food security by obtaining nutritious FAVs within a short period throughout the year.

## Graphical abstract

The levels of vitamin C and E, and β‐carotene in hydroponic and soil‐based tomatoes, spinach, and strawberries were analyzed. Vitamin C and E were significantly higher in hydroponically grown tomatoes and strawberries. β‐carotene was significantly higher only in the hydroponic spinach. Hydroponic system does not contribute to the introduction of naturally absent nutrients in a crop such as β‐carotene in strawberries.

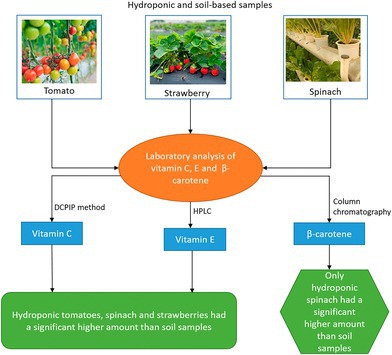

**Abbreviations**
ANOVA: analysis of variance
BHT: butylated hydroxytoluene
DCPIP: dichlorophenolindophenol
FAVs: fruits and vegetables
HFS: hydroponic farming system
HPLC: high performance liquid chromatography
LC: liquid chromatography
LCYb: lycopene β‐cyclase
Mg/FW: milligrams per fresh weight
NCDs: non‐communicable diseases
NFT: nutrient film technique
KOH: potassium hydroxide
RDA: recommended dietary allowance
SPSS: Statistical Package for Social Sciences
SSA: sub‐Saharan Africa
TCA: trichloroacetic solution
UV–VIS: ultraviolet–visible light

## 1 Introduction

Fruits and vegetables (FAVs) have vitamins and minerals in considerable quantities that might otherwise be limited in animal‐based food sources (Kyriacou and Rouphael 2018). This has led to recommendations such as increased consumption of FAVs for consumers to achieve the recommended dietary allowances (RDA). The antioxidant capacity in FAVs has been linked to a reduction in non‐communicable diseases (NCDs) (del Rio‐Celestino and Font 2020; Dulińska‐Litewka et al. 2021) that are soaring high in both developed and developing countries, especially among the poorest individuals and households (Manderson and Jewett 2023). NCDs refer to usually chronic health conditions that are non‐transferable from one person to another (Piovani et al. 2022). Consumption of healthy foods such as FAVs that can be grown by consumers on small plots, backyards, and in recyclable materials for individual and household use, are considered one of the modifiable behavioral factors to manage and prevent the progression of NCDs (Fussy and Papenbrock 2022; Ramkumar et al. 2024). Even though the amount of nutrients varies per FAV, their presence in the diet is significant in keeping the doctor away, given an increase in the portion size, consumption frequency, and daily incorporation in the diet (Blas et al. 2019). Of interest in this study are β‐carotene, vitamins C and E nutrients which must be counted among the antioxidant compounds necessary for intake because of their anti‐cancer and anti‐inflammatory roles, the fight against heart and eye diseases, among other metabolic syndrome diseases (del Rio‐Celestino and Font 2020).

Vitamin C is a major antioxidant that is necessary for the functionality of the cells in the human body (Tan et al. 2020). It is an essential nutrient which has to be obtained through the diet for it to function in the body. It is found in foods such as liver, kidney, and fortified foods. It is commonly found in fruits such as oranges and citrus fruits, strawberries and papaya, and in vegetables such as spinach, kale, broccoli, and cabbage. A limited or lack of intake of this nutrient can easily lead to a deficiency; hence, scurvy, which manifests with symptoms that range from general body weakness to bleeding of gums. An RDA of 75 mg in females and 90 mg in males is required to prevent it and is sufficient for individuals to meet the bodily nutritional needs in (NIH 2021a).

Unlike vitamin C, vitamin E is a fat‐soluble nutrient that is found in the fatty regions in the body. However, its provision through the diet is necessary to avoid complications associated with its deficiency. It is a group of eight fat‐soluble compounds, among which only the α‐tocopherol form is used in the human body (Lee et al. 2018). It is found in foods such as egg yolk and shellfish. It is commonly found in fruits such as avocado, mango, papaya, and kiwi, and in vegetables such as spinach, Swiss chard, and asparagus. The National Institutes of Health recommends that an adult person, both male and female, should consume 15 mg (22.1 International Units) of this nutrient daily to meet the bodily nutritional needs (NIH 2021b). It functions to prevent neurological abnormalities, enhance immunity through protection of polyunsaturated fatty acid membrane from oxidation, and regulation of production of reactive oxygen species. It has anti‐inflammatory and anti‐proliferative effects, and it prevents atherosclerosis, which is associated with cancer and loss of vision (Dulińska‐Litewka et al. 2021).

β‐carotene, on the other hand, is the major precursor of vitamin A, which is responsible for the yellow/orange color in foods (Murage 2020). It is also known for its antioxidant property in cancer prevention. It is found in foods such as egg yolk, shellfish, and nuts and seeds such as almond and sunflower seeds, respectively. It is commonly found in fruits such as mango, papaya, and apricot, and in vegetables such as carrots, pumpkin, spinach, and kales. An RDA of 2–7 mg/100 g is deemed necessary to help balance the retinol supply in the body; hence, prevent inadequacy (Nambafu et al. 2021).

It has been hypothesized that the methods used in the production of FAVs, nutrients applied, and activities carried out in different stages of crop growth have a significant effect on the accumulation of nutrients in FAVs (Gashgari et al. 2018; Rahman and Zhang 2018). This has been stated positively in support of the hydroponic farming system (HFS) (Rouphael et al. 2018; Tan et al. 2020). The HFS refers to the soilless farming method in which crops grow in an inert media instead of soil, and the roots are immersed in a nutrient‐rich solution. The environmental conditions such as temperatures and humidity are usually controlled using timers, pumps, and facilities such as greenhouses and indoor equipment, and generally require less water (Kannan et al. 2022). On the other hand, the conventional soil farming system refers to the method in which crops are grown directly in soil, outdoor in an open field/space, and under the natural environmental factors such as temperature, humidity, rainfall, etc. Sometimes irrigation systems are employed, and there is active application of organic or inorganic nutrients (Fussy and Papenbrock 2022). In the past decades, FAVs in Kenya have been grown in soil‐based environments which are currently being affected by climate variability and climate change (Boliko 2019; Rouphael et al. 2018). With the foreseen exhaustion of natural resources and reduction of soil quality and integrity, crops may no longer get enough nutrients in a natural manner for growth to produce sufficient output with the required nutrients that meet the health and nutritional needs of consumers (Aires 2018). This is even made worse with the application of ineffective inorganic chemicals that compromise the integrity of the soil to feed crops because of the accumulation of residues (Rahman and Zhang 2018).

The above concerns seem to be solved by HFS. This system has several advantages, especially in Sub‐Saharan African (SSA) countries such as Kenya that continue to face the impacts of climate change. The immersion of the roots of food crops in water promotes efficient water utilization, reduces crop stress, and promotes direct uptake of nutrients, which contributes to the growth and production of high‐quality foods in large quantities throughout the year (Aires 2018; Shubham and Shrimanth 2020). The chances of crop failure in this system are therefore lower as compared to the soil‐based system. The set‐up of HFS makes food production in urban areas possible, reducing dependency on arable land to produce foods for all (Bhattarai and Adhikari 2023). This could contribute to food and nutrition security in the areas that often face food inaccessibility due to high costs of fresh nutritious foods. HFS also provides entrepreneurial opportunities to otherwise unemployed youths, hence breaking the vicious cycle of parental dependency and intergenerational poverty in the long run (Amusan and Adeleke 2023).

However, it is also worth noting that the HFS has a few disadvantages that could make some farmers hesitate to install it. It requires a huge initial financial investment and technical know‐how, which most farmers might be lacking (Mishra et al. 2024). The system is heavily reliant on the constant supply of energy, which is a big challenge in SSA due to the high electricity costs and constant power outages (Mishra et al. 2024). From a sustainability perspective, some HFS pose environmental concerns due to the dependency on synthetic nutrients to make the nutrient solution in which the crops are immersed (Fussy and Papenbrock 2022). This calls for proper management skills from each farmer, which might vary from one person to another (Rouphael et al. 2018). This insinuates that inappropriate usage of nutrients, i.e., usage of amounts lower or higher than the required, might not be favorable to crops, hence affecting the accumulation of nutrients in food crops (Aires 2018). Since the employment of HFS in countries such as Kenya is in its introductory stage, the assurance of safety and quality of food crops is largely dependent on the knowledge, skills, and practices of the farmers. Lastly, the perception of consumers about hydroponically produced foods is still low as they are still familiarizing themselves with the foods that are produced in such “untraditional” way as compared to the soil‐based foods (Nekesa et al. 2023).

Keeping in view the above background associated with the HFS, and its novelty in Kenya, it is essential to determine the nutrient levels in FAVs grown in this system since the demand for nutritious foods to curb NCDs is rising exponentially. It is in this regard that this study focused on the analysis and comparison of the levels of β‐carotene, vitamin C, and E in hydroponic and soil‐based spinach, tomato, and strawberry.

## 2 Materials and Methods

### 2.1 Study Site

The study was carried out in Kiambu County in Kenya since it had the highest number of hydroponic farms using nutrient film technique (NFT) than other counties in the country. Kiambu County is located in Central Kenya in the north of Nairobi between latitude 00°25′ and 10°20′ South and longitude 36°31′ and 37°15′ East (Bolo et al. 2023).

### 2.2 Study Design

A cross‐sectional study design with an analytical component was used to gather data. The analytical part involved a laboratory analysis to determine the level of β‐carotene, vitamin C, and E in spinach, tomatoes, and strawberries.

### 2.3 Sampling

#### 2.3.1 Sampling Unit

The sampling unit was the hydroponic farms using NFT and soil‐based farms with spinach and/or tomatoes and/or strawberries. A pre‐visit was made to establish the available farms with the selected FAVs under study.

#### 2.3.2 Sample Size

Out of the 10 identified hydroponic farms with NFT, only six farms were growing spinach, tomatoes, and/or strawberries at the time of data collection. The roots of the plants were immersed in the nutrient solution in pipes or troughs. The water was pumped to the grow channels where the roots rested and absorbed nutrients. The solution flowed back into the reservoir for reuse. In the case of tomatoes, larger pipes and larger reservoirs were used because of the size of the plants. From the six farms, two farms were excluded from the study because of denial of permission from the management to collect samples for analysis. Only three farms had all the FAVs under study in their last stage of maturity. One farm had only spinach and was hence included in the study. Therefore, a total of four hydroponic farms using NFT were included in the study. Hence, three samples of tomatoes, three samples of strawberries, and four samples of spinach were collected from the hydroponic farms. From the soil‐based farms, no single farm had all the FAVs required for the study. The farms were open fields that were rain‐fed and had no controlled environment by the farmers. Therefore, spinach, tomatoes, and strawberries that were in their last stage of maturity were collected from nine separate farms, where three samples of spinach, three samples of tomatoes, and three samples of strawberries were obtained from the selected soil‐based farms.

#### 2.3.3 Sampling Procedure and Sample Collection

The samples were collected in early September when the harvest of strawberries and tomatoes from the soil‐based farms was ongoing. This applied for spinach as well since it is grown year‐round in Kiambu County because of the mild climate. The hydroponic crops were not dependent on seasons since their environment is controlled by the farmer. The samples were randomly selected both in the hydroponic farms and soil‐based farms. Each sample comprised parts picked from different points in the farms. With spinach, mature leaves were picked from several stems from the entrance, middle point, and the end of the farms then mixed to make a sample. Ripe tomatoes (red in color) were picked in the like manner and combined to make a sample per farm. Similarly, ripe strawberries were selected and put together in a punnet to make a sample. Each sample was put in a clean new shopping bag which was labeled with three letters according to the farming system from which the sample was obtained, the name of the fruit or vegetable being handled, and the last letter represented the area in which the farm was located. Strawberries were first put in punnets before being placed in the shopping bags to avoid being bruised in case of poor handling. The samples were taken directly to the laboratory in the University of Nairobi, Kenya, for analysis within 24 h to avoid deterioration in the quality and quantity of the nutrients.

#### 2.3.4 Sample Preparation

**FIGURE 1:**
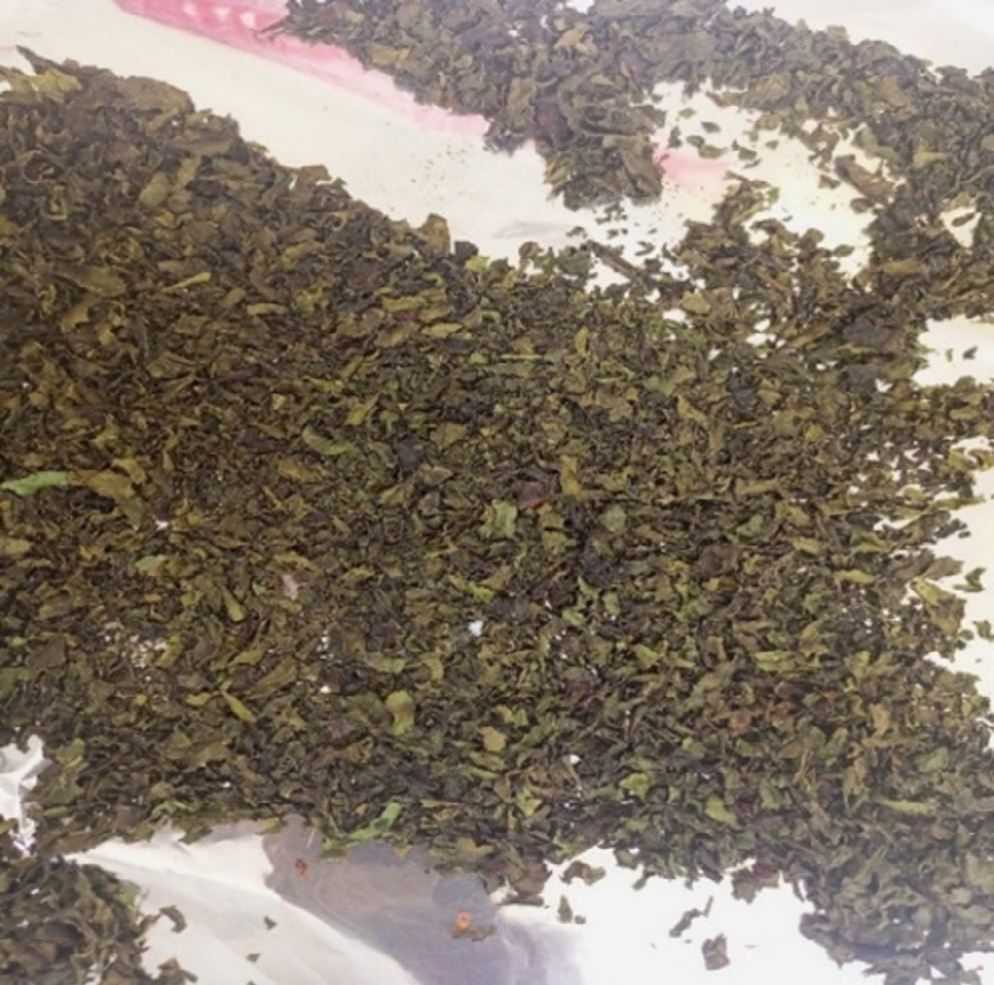
Dried spinach from the air dryer after 24 h.

**FIGURE 2:**
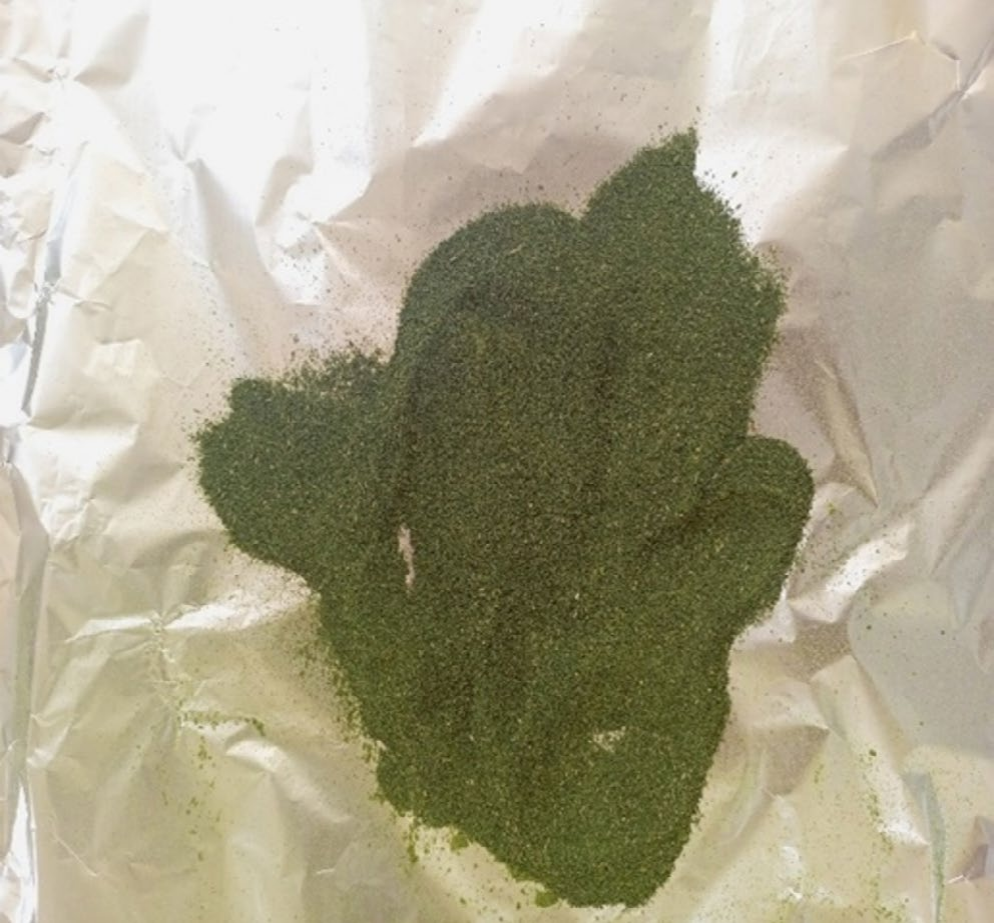
Powdered spinach after grinding using a blender.

**FIGURE 3:**
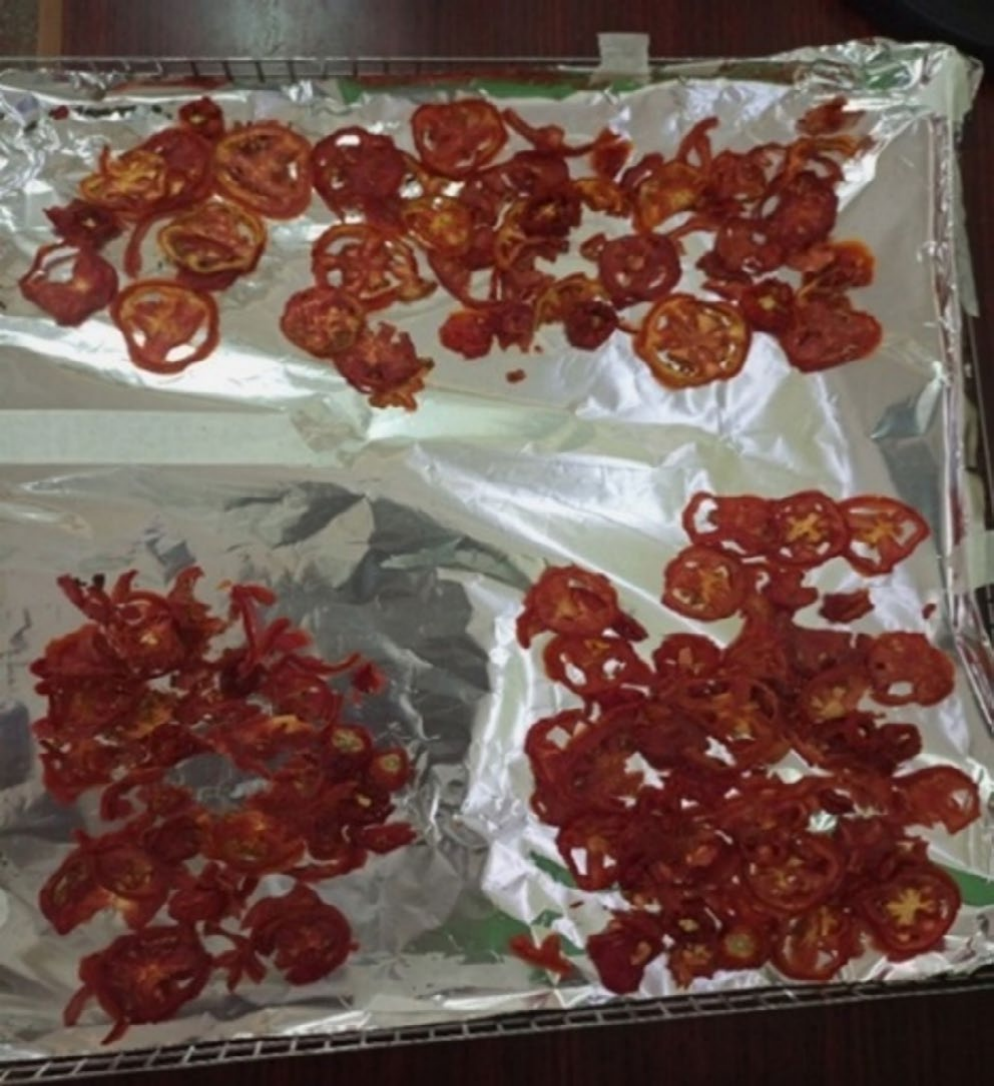
Dried tomatoes from the oven after 24 h.

**FIGURE 4:**
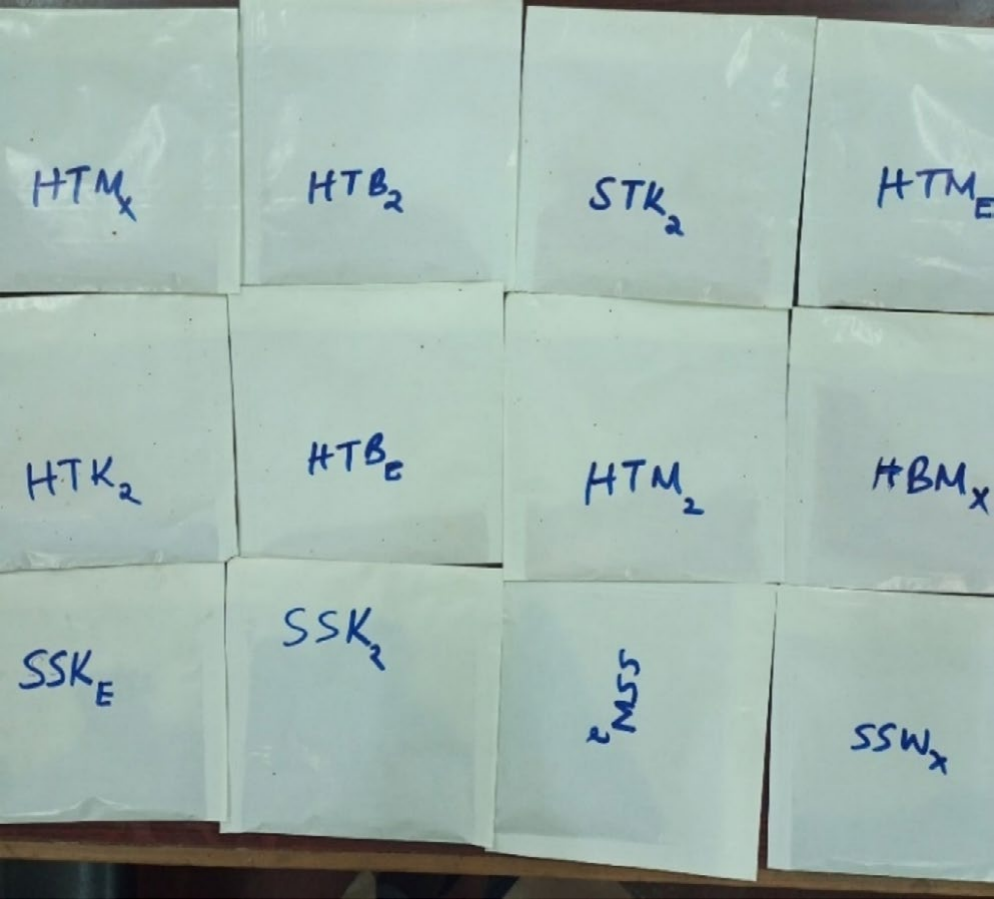
Packaged samples of spinach, tomatoes, and strawberry in powder form.

Samples from each farm were chopped into small pieces (2–3 cm in diameter) on clean steel trays without being subjected to any form of treatment such as washing. The pieces were mixed by hand to achieve homogeneity. 2 g of each sample were taken for moisture determination. For the analysis of vitamin C and β‐carotene, 50 g of each sample were set aside from the batch for use. Due to the requirement to analyze vitamin E in the samples away from the Food Chemistry laboratory at the University of Nairobi, Kenya, the samples were dried to avoid deterioration in quality. The samples were put on aluminum foils which were then placed in the clean steel trays and labeled. The trays were put in the air dryer at 75°C for 24 h. The dry samples were then separately blended to powder form, packed in the waterproof packing bags and taken to Jomo Kenyatta University of Agricultural Technology for vitamin E analysis. The air‐dried spinach from a hydroponic farm is shown in Figure 1. A blender was used to achieve homogeneity and to get the powdered form as shown in Figure 2. Figure 3 shows tomatoes from three soil‐based farms after being dried for 24 h in the air dryer while Figure 4 shows the packaged spinach, tomatoes, and strawberry in powder form. The letters on the packaging materials are unique to the farming system from which the samples were obtained where the first letter H represented the hydroponic system while the first S represented the soil‐based system. The second letter was unique to the fruit or vegetable being studied where S represented spinach, T represented tomato, and B for strawberry. The last letter represented the areas from which the samples were obtained.

### 2.4 Analytical Methods

#### 2.4.1 Analytical Chemicals

The chemicals used in the analysis of vitamin C were Trichloroacetic (TCA) solution, which was manufactured in France and branded as Biochem Chemopharma, and Dichlorophenolindophenol (DCPIP) reagent, which was manufactured in Kenya by Unilab Kenya Limited. In the analysis of β‐carotene, petroleum spirit was manufactured in India by Loba Chemie PVT. LTD and branded as Loba Chemie. Acetone was also manufactured in India by Span Chemie (SC). All reagents used in the analysis of vitamin E, including ethanol, potassium hydroxide (KOH), hexane stabilized with 0.2% butylated hydroxytoluene (BHT), and BHT‐supplemented ethanol (0.1%), were of analytical grade and manufactured by Sigma‐Aldrich in Spain.

#### 2.4.2 Vitamin C Analysis

The vitamin C content in spinach, tomatoes, and strawberries was determined using the DCPIP method (Abidin and Ikhwan 2024). 5 g of each sample was mixed with 60 mL of 5% TCA solution. The mixture was filtered into a 100 mL volumetric flask, which was filled to the mark with 5% TCA solution. 10 mL of the solution was titrated with standardized DCPIP reagent in duplicates. The results were obtained in milligrams per 100 g of the sample's fresh weight (mg/100gFW).

#### 2.4.3 β‐Carotene Analysis

Β‐carotene was analyzed using the column chromatography method (Amalya and Sumathy 2014). 5 g of each sample were taken and the color was extracted using a pestle and mortar while adding small portions of acetone until the residue was colorless. All the extracts were combined into a 100 mL volumetric flask. 25 mL of the extract were drawn from the 100 mL volumetric flask and evaporated to dryness using a rotary evaporator at 60°C. The rotary evaporator was manufactured in Germany by Janke & Kunkel GmbH u. CoKG and branded as IKA (IKA‐Werk 7813 Staufen). 1 mL of petroleum spirit was added to the evaporated sample to dissolve β‐carotene, which was then eluted through a packed column chromatography. The yellow/orange color was received in a 25 mL volumetric flask, then absorbance was read at 450 nm. The β‐carotene level was calculated from a β‐carotene standard curve and the results were presented in mg/100gFW.

#### 2.4.4 Vitamin E Analysis

Vitamin E in spinach, tomatoes, and strawberry was measured as α‐tocopherol by High Performance Liquid Chromatography (HPLC) (Irakli et al. 2016). The HPLC (SHIMADZU 20 ad fitted with SPD 20A detector) was manufactured in Kyoto, Japan, by Shimadzu Corporation. Duplicate tissue samples (2.0 g) were homogenized with 4 mL of 95% ethanol and 1 mL of 50% KOH. The mixtures were saponified by heating in a 70°C water bath for 15 min and then cooled in an ice bath. Fat‐soluble vitamins were extracted with 1 mL hexane containing 0.2% BHT, and a 1 mL aliquot of the hexane layer was evaporated under nitrogen. Saponification, extraction, and evaporation procedures were performed under yellow light. Samples were then reconstituted with 0.25 mL ethanol containing 0.1% BHT. A Shimadzu LC‐20 ad Series liquid chromatograph equipped with a 250 × 4.0 mm stainless steel Octadecylsilane (ODS) reversed‐phase column was used to quantify α‐tocopherol as measures of vitamin E. The mobile phase was a 96:4 methanol to water ratio for α‐tocopherol detection and separation at a flow rate of 1 mL/min. The α‐tocopherol was monitored at 285 nm wavelength on an ultraviolet/visible light (UV–VIS) detector (Shimadzu SPD‐20A). External standards were compared to sample extracts for determination of vitamin E concentrations.

### 2.5 Statistical Analysis

Data was analyzed using Statistical Package for Social Sciences (SPSS) (version 25). The levels of β‐carotene, vitamin C, and E in each sample were expressed as means and standard deviations. One‐way analysis of variance (ANOVA) and post‐turkey test were used to determine the statistical difference in the levels of β‐carotene, vitamin C, and E samples obtained from hydroponic and soil‐based farms. Independent sample T‐test was used to determine the significant difference between the samples obtained from the hydroponic and soil‐based systems. The level of significance was determined at 5%.

## 3 Results

### 3.1 Level of Vitamin C in Hydroponic and Soil‐Based Spinach, Tomatoes, and Strawberry

The results of the level of vitamin C in tomatoes, spinach, and strawberries from hydroponic and soil‐based farms are presented in Table 1. Overall, the level of vitamin C in the hydroponically grown tomatoes was significantly higher (*p* = 0.008) than that of the soil‐based samples. This was contributed to by the slightly high amounts of the nutrient in tomatoes from each hydroponic farm sampled. However, a post‐Tukey test revealed significant variations (*p* < 0.05) in the nutrient levels in the samples obtained from the three hydroponic farms. With regard to spinach, there was no significant difference in the levels of vitamin C in the samples obtained from the hydroponic and soil‐based systems (*p* = 0.248). Nevertheless, the samples with the highest levels of vitamin C (112.81 ± 0.28 mg/100gFW and 93.61 ± 0.24 mg/100gFW) were obtained from the hydroponic farms as opposed to the soil‐based samples. Similar to the results of the post‐Tukey test of tomatoes, there was a significant difference in the levels of vitamin C in the samples obtained from two hydroponic farms in Banana and Muthiga (*p* < 0.05). Notably, the level of vitamin C in hydroponically grown strawberries was significantly higher than that of the soil‐based samples (*p* = 0.037). A significant difference in the post‐Tukey test was also observed in the level of vitamin C in the strawberries obtained from the three hydroponic farms and also in the soil‐based farms.

**TABLE 1:** The level of vitamin C in spinach, tomatoes and strawberry obtained from selected hydroponic and soil‐based farms in Kikuyu and Kiambu sub‐counties.

Sample name	Hydroponic‐based farms		Soil‐based farms		*p‐value*
	Mean ± SD (mg/100gFW)		Mean ± SD (mg/100gFW)		
	Area	Vitamin C	Area	Vitamin C	
Tomato	Muthiga	35.92 ± 0.05^a^	Wangige	24.22 ± 0.23^a^	
	K‐Road	40.70 ± 0.03^b^	Kikuyu	22.21 ± 0.18^b^	
	Banana	31.50 ± 0.41^c^	Ndumbuini	28.48 ± 0.04^c^	
	Overall level	36.04 ± 0.16		24.81 ± 0.15	0.008
Spinach	Ruiru	112.81 ± 0.28^a^	Wangige	81.21 ± 0.13^a^	
	Muthiga	53.01 ± 0.09^b^	Kikuyu	63.91 ± 0.14^b^	
	Banana	53.70 ± 0.07^b^	Ndumbuini	89.34 ± 0.08^c^	
	K‐Road	93.61 ± 0.24^c^			
	Overall level	78.28 ± 0.17		78.15 ± 0.12	0.248
Strawberry	Ndumbuini	80.84 ± 1.74^a^	Ruiru	85.41 ± 0.59^a^	
	Muthiga	85.01 ± 0.03^b^	Ndumbuini	87.07 ± 0.06^b^	
	K‐road	82.01 ± 0.07^c^	K‐Road	62.51 ± 0.23^c^	
	Overall level	82.29 ± 0.61		78.33 ± 0.29	0.037

*Note:* All analytical data are the means of the duplicate measurements of each sample obtained from farms in different areas in kikuyu and Kiambu sub‐counties. *p*‐values are for independent sample T‐test between hydroponic and soil‐based samples. The different superscripts in a column of hydroponic and soil‐grown FAVs show a significant difference at 5% in the post‐Tukey test.

### 3.2 Level of Vitamin E in Hydroponic and Soil‐Based Spinach, Tomatoes, and Strawberry

Table 2 presents the results of the level of vitamin E in tomatoes, spinach, and strawberries grown in hydroponic and soil‐based systems. The results show that the levels of vitamin E in the hydroponically grown tomatoes, spinach, and strawberries were significantly different from the soil‐based samples (*p* = 0.050, *p* = 0.000 and *p* = 0.041, respectively). Regarding tomatoes, the levels of vitamin E in the samples obtained from hydroponic farms were slightly higher than that in the soil‐grown samples. However, there was a great disparity in these quantities of samples from each hydroponic farm, which accounted for the significant difference in the post‐Tukey test. It was remarkable that the overall level of vitamin E in the hydroponically grown spinach (21.50 ± 1.13 mg/100gFW) was five times higher than in the soil‐based samples. A post‐Tukey test revealed a significant difference in the level of vitamin E in the spinach samples obtained from the three hydroponic farms. While the level of vitamin E was generally low in all strawberry samples, all the samples from hydroponic farms had higher levels of the nutrient compared to the samples from the soil‐based farms. Unlike the post‐Tukey test results of hydroponically grown tomatoes and spinach, these quantities from the samples in each farm were not significantly different from each other.

**TABLE 2:** The level of vitamin E in spinach, tomatoes, and strawberries obtained from selected hydroponic and soil‐based farms in Kikuyu and Kiambu sub‐counties.

Sample name	Hydroponic‐based farms		Soil‐based farms		*p*
	Mean ± SD (mg/100gFW)		Mean ± SD (mg/100gFW)		
	Area	Vit E	Area	Vit E	
Tomato	Muthiga	5.88 ± 0.03^a^	Wangige	10.10 ± 0.31^a^	
	K‐Road	22.67 ± 2.81^b^	Kikuyu	5.50 ± 0.43^b^	
	Banana	10.91 ± 0.05^c^	Ndumbuini	10.10 ± 0.08^a^	
	Overall level	12.62 ± 0.97		8.57 ± 0.27	0.050
Spinach	Ruiru	23.82 ± 0.28^a^	Wangige	5.46 ± 0.28^a^	
	Muthiga	25.10 ± 1.60^b^	Kikuyu	4.11 ± 0.43^a^	
	Banana	22.41 ± 1.68^a^	Ndumbuini	4.10 ± 0.23^a^	
	K‐Road	14.67 ± 0.97^c^			
	Overall level	21.50 ± 1.13		4.56 ± 0.31	0.000
Strawberry	Ndumbuini	7.03 ± 1.01^a^	Ruiru	3.64 ± 0.15^a^	
	Muthiga	5.40 ± 0.58^a^	Ndumbuini	4.71 ± 0.47^a^	
	K‐road	6.87 ± 0.11^a^	K‐Road	6.76 ± 0.07^b^	
	Overall level	6.43 ± 0.57		5.04 ± 0.23	0.041

*Note:* All analytical data are the means of the duplicate measurements of each sample obtained from farms in different areas in Kiambu County. *p*‐values are for independent sample T‐test between hydroponic and soil‐based samples. The different superscripts in a column of hydroponic and soil‐grown FAVs show that there was a significant difference (*p* ≤ 0.05) in the post‐Tukey test.

### 3.3 Level of β‐Carotene in Hydroponic and Soil‐Based Spinach, Tom,atoes and Strawberry

The results of the levels of β‐carotene in tomatoes, spinach, and strawberries in hydroponic and soil‐based farms are presented in Table 3. There was a significant difference in the level of β‐carotene in hydroponic tomatoes and spinach compared to the soil‐based samples (*p* = 0.050 and *p* = 0.000, respectively). Whereas the levels of vitamin C and E were significantly higher in hydroponically grown tomatoes, as shown in Tables 1 and 2, the level of β‐carotene was significantly lower than that of the soil‐grown tomatoes. There were no significant differences in the levels of β‐carotene in tomatoes obtained from the three hydroponic farms in the post‐Tukey test. With respect to spinach, the overall level of β‐carotene in the samples obtained from hydroponic farms (4.42 ± 0.32 mg/100gFW) was two times higher than that of the soil‐grown spinach. Likewise, there were no significant differences in the post‐Tukey test in the level of β‐carotene in samples obtained from the three hydroponic farms. The most striking observation was that no β‐carotene was detected in both hydroponic and soil‐grown strawberry samples.

**TABLE 3:** The level of β‐carotene in spinach, tomatoes, and strawberry obtained from selected hydroponic and soil‐based farms in Kikuyu and Kiambu sub‐counties.

Sample name	Hydroponic‐based farms		Soil‐based farms		*p*
	Mean ± SD (mg/100gFW)		Mean ± SD (mg/100gFW)		
	Area	Β‐carotene	Area	Β‐carotene	
Tomato	Muthiga	2.64 ± 0.03^a^	Wangige	3.38 ± 0.02^a^	
	K‐Road	2.42 ± 0.09^a^	Kikuyu	4.23 ± 0.08^b^	
	Banana	2.55 ± 0.01^a^	Ndumbuini	4.59 ± 0.08^b^	
	Overall level	2.54 ± 0.04		4.06 ± 0.06	0.050
Spinach	Ruiru	3.85 ± 0.07^a^	Wangige	2.22 ± 0.03^a^	
	Muthiga	4.61 ± 0.06^a^	Kikuyu	2.14 ± 0.03^a^	
	Banana	4.45 ± 0.03^a^	Ndumbuini	1.26 ± 0.09^a^	
	K‐Road	4.77 ± 0.01^a^			
	Overall level	4.42 ± 0.32		1.87 ± 0.05	0.000
Strawberry	Ndumbuini	ND	Ruiru	ND	
	Muthiga	ND	Ndumbuini	ND	
	K‐road	ND	K‐Road	ND	

*Note:* All analytical data are the means of the duplicate measurements of each sample obtained from hydroponic and soil‐based farms. *p*‐values are for independent sample T‐test between hydroponic and soil‐based samples. The different superscripts in a column of the hydroponic and soil‐based FAVs show that there was a significant difference (*p* ≤ 0.05) in the post‐Tukey test. ND means not detected.

## 4 Discussion

The association of HFS with high nutrient density in food crops has also been observed in this study. It is worth noting that besides the high accumulation of nutrients, the crops take a considerably less period of growth, which is desirable to curb hunger and food insecurity, especially in low‐nd middle‐income countries such as Kenya. In the past, consumers have been guided to consume organically produced foods because of a substantially higher nutritional content. From the findings of this study, HFS could provide synergy to this narrative as it offers even better outcomes (Aires 2018). Fundamentally, the traditional soil‐based method of growing crops in Kenya can therefore be backed up with such efficient methods with less fear of facing the negative effects of climate change and variability, and deprivation of natural resources.

The results of this study showed that the levels of vitamin C in tomatoes and strawberries were significantly higher than in the ones obtained from the soil‐based farms. This was consistent with the studies by Fevria et al. (2021), Kumari et al. (2018) and Tõnutare and Keert (2014) where the nutritional value of several food crops grown hydroponically was found to have higher amounts of nutrients compared to the soil‐grown foods. Firstly, this study showed that the level of vitamin C was consistently high in the tomatoes sampled from the three hydroponic farms. As a matter of fact, the overall amount was numerically higher (36.04 mg/100gFW) than those that have been reported in some previous studies. To mention just a few results from different studies, the levels ranged from as low as 8.9–19.3 mg/100gFW (Agbede et al. 2018; Cruz‐rus et al. 2012; Hallman et al. 2013). The high levels of vitamin C in the hydroponic tomatoes could be attributed to the well‐conditioned environment in the hydroponic farms where the temperature is regulated and the minerals are constantly supplied in the water to the crops. From the agricultural point of view, these are the vital requirements for proper crop growth and specifically for the accumulation of vitamin C in FAVs (Agbede et al. 2018).

With reference to strawberries, hydroponically grown samples had significantly higher amounts of vitamin C than the soil‐based samples. The findings were consistent with the findings in the study by Treftz and Omaye (2015) where hydroponically grown strawberries had 74% higher vitamin C content than the soil‐based strawberries. More evidence is provided from the study by Falah et al. (2020) which reported that hydroponically grown strawberries had significantly higher content of vitamin C (*p* < 0.05) compared to the soil‐based strawberries despite the varieties used. Similarly, the study by Zhong et al. (2017) recorded a range of 37.96–52.48 mg/100gFW of vitamin C in four varieties of soil‐based strawberries which is lower than the overall level of hydroponically grown strawberries found in this study. Even after compartmentalizing soil‐based strawberries into conventional and organic farming methods, the results from this study were still meritoriously higher by 26 mg/100gFW and 13 mg/100gFW, respectively (Tõnutare and Keert 2014). Overall, the high level of vitamin C in the hydroponic strawberries in this study could be because of the constant nutrients channeled to the plant roots and the proper drainage which is necessary for strawberries to efficiently take in nutrients for growth (Falah et al. 2020). The traditional soil‐based method capitalizes on watering strawberries from above which makes the water insufficient in the roots. Additionally, in the event water is irrigated close to the ground, some soils have poor drainage leading to clogging which limits the required amount of water to reach the plant roots (Bhardwaj et al. 2024). Besides, the water could have fewer minerals that the plants require to grow especially when the aim is to just water them rather than delivering the nutrients.

Whereas the level of vitamin C is expected to be generally high in spinach, analyzing it based on the farming system elicits interesting scenarios. Firstly, even though there was no significant difference in the overall levels of this nutrient in hydroponic and soil‐grown spinach, two hydroponic farms produced the highest concentrated spinach with vitamin C compared to the samples obtained from the soil‐based farms in the current and previous studies. This observation is supported by the evidence from the study by Zeng (2013) that reported the highest level of vitamin C (89.0 ± 0.1 mg/100gFW) in the soil‐grown spinach, which is plainly lower than those recorded from the two hydroponic farms in this study. Furthermore, the physical examination of the samples from the two hydroponic farms aided a relevant explanation of the commendable level of vitamin C in the spinach samples. The broad‐sized and tender leaves embedded a darker green color, which is commonly linked to the presence of vitamins, including vitamin C, in leafy vegetables. This observation was incomparable to the soil‐grown spinach. The lower levels of vitamin C in the spinach obtained from the other two hydroponic farms could have been due to the poor management of the vegetable, which greatly contributes to lower accumulation of nutrients in food crops. This agrees with the established scientific knowledge that misappropriation of minerals required by the crops leads to poor nutrition in crops, hence a low nutrient density (Bhardwaj et al. 2024). On this account, it should be considered that the value indicated in this study as the overall level of vitamin C in hydroponic spinach might not be the exact reflection of the potential of hydroponically grown spinach to accumulate vitamin C.

In the second scenario, there was a small variation in the levels of vitamin C in spinach samples obtained from the three soil‐based farms. This could be due to mastery of the art of soil‐farming techniques to grow spinach in the soil system, as this has been used in Kenya since antiquity. As a consequence, farmers are aware of the harsh conditions that their vegetables are subjected to; thus, they successfully work with the biotic conditions to the advantage of the crops. The best possible outcome is, therefore, a relatively uniform accumulation of nutrients in the soil‐grown spinach within the same region (Das et al. 2020). Anyhow, this can also be attained by hydroponic farmers, considering that they have an upper hand in controlling the harsh abiotic factors that are majorly a cause of crop failure and nutritional inadequacy in food crops.

In the context of vitamin E, hydroponically grown tomatoes, spinach, and strawberries stood out with higher levels compared to the soil‐based samples. This was comparable to the findings from the studies by Tõnutare and Keert (2014) and Zhong et al. (2017). The most relevant explanation for these results is drawn from the comments made by Aires (2018) that vitamin E is the exceptional antioxidant that has the highest rate of accumulation in hydroponically grown food crops since the system improves the antioxidant activity in the crops. For this reason, there is an increased accumulation of the nutrient to even five times more than in soil‐based systems as observed in spinach samples in this study. The results of this study are reproducible also with the focus on other hydroponically and soil‐based crops such as lettuce and basil despite the varieties. This is evidenced by the evaluation carried out by Buchanan and Omaye (2012) on the level of vitamin E in three varieties of lettuce grown hydroponically and in soil systems. It was concluded that hydroponically grown lettuce had significantly higher levels of vitamin E (*p* < 0.05) in all three varieties compared to soil‐based lettuce. Bearing in mind the time of growth, the results of Sgherri et al. (2010) also showed that the level of vitamin E in a 35‐day‐old hydroponic basil was much higher compared to a 35‐day‐old soil‐grown basil. In addition, they reported that crops grown in the former system were also found to be healthier and appealing in color compared to those obtained from soil‐based farms. Due to limited studies that compare the amount of vitamin E in hydroponic and soil‐grown tomatoes and spinach, the above findings on other FAVs could be extrapolated to inform on the variation in the level of vitamin E in these two systems.

Distinct from a number of studies that have reported high levels of β‐carotene content in hydroponically grown FAVs (Kong and Nemali 2023; Verdoliva et al. 2021), the findings of this study were inconsistent, especially on tomatoes. Nonetheless, the overall level of β‐carotene in hydroponic tomatoes in this study was above average for the amount of β‐carotene expected in tomatoes since they are known for high concentrations of lycopene (Murage 2020). On the other hand, the abnormally high level of β‐carotene in the soil‐based tomatoes could be associated with the overexpression of lycopene β‐cyclase (LCYb) in the varieties of tomatoes grown by farmers and the overutilization of hormone ethylene for faster ripening of tomatoes, which is associated with the accumulation of β‐carotene (Diretto et al. 2020). The majority of the soil‐based farmers are economically motivated to practice this in order to supply their produce and get a higher profit (Chikkanna et al. 2022). In the end, the level of β‐carotene in tomatoes is high but not as a result of good farming practices. This is detrimental since overconsumption of FAVs with hormonal residues predisposes consumers to diseases associated with the accumulation of chemical contaminants in the body tissues, hence growth defects in babies and even death (Thompson and Darwish 2019).

With reference to spinach, it was interesting to note that the high level of β‐carotene in the hydroponic‐based samples unmatched the common belief that soil‐based food crops undergo better carotenogenic processes than the latter because of better exposure to sunlight and relatively high temperatures. While this could be scientifically true, the findings of this study suggest that there might be more elements other than favorable abiotic factors that are needed to promote carotenogenic processes in food crops. This might include the variety of the crop since it plays an important role in the survival of the crop at a given intensity of sunlight and temperature (Anwar et al. 2021). Another reason could be the difference in the management practices applied in the field such as constant nutrient supply to the crop (Mutschlechner et al. 2022). Sadly, the hydroponic system does not contribute to the introduction of naturally absent nutrients in a crop such as β‐carotene in strawberries. This is to imply that the system only promotes the accumulation of already available nutrients in the crop which achieves a higher nutrient density compared to the soil‐based system.

The significant variation in the levels of vitamin C and E in tomatoes and spinach sampled from different hydroponic farms was not peculiar to this study. The results were comparable to the study by Buchanan and Omaye (2012) and Olubanjo and Alade (2018) that reported a wide difference in vitamin E levels in lettuce and pepper grown in different hydroponic farms in the United States of America and in Nigeria, respectively. This is simply because farmers tend to carry out crop management activities uniquely in their farms out of knowledge and/or constriction of the required resources (Shubham and Shrimanth 2020). Such activities include application of fertilizers and chemicals at different levels, use of different seed varieties, control of pests, insects, and diseases, use of water with different quality levels, among others. It is therefore expected that improper execution of these activities potentially leads to poor nutrition of the crop, hence inadequate nutrient accumulation. Nonetheless, there is confidence that farmers in Kenya are still familiarizing themselves with the hydroponic system for the growth of different crops, hence the benefit of trial and error. This is to infer that in the few years to come, with adequate training and constant practice on the farms, the margin of difference in the nutrient level in similar crops grown by different farmers using hydroponic systems might be closed. This will result in a proportionally uniform level of nutrients regardless of the hydroponic farm from which the crops are obtained. Anyway, hydroponically grown crops undergo less oxidative stress in search of minerals for growth compared to soil‐based crops, hence more nutrient accumulation than the latter (Jha et al. 2023; Verdoliva et al. 2021).

## 5 Conclusion

The findings of this study revealed that consumers are better placed with hydroponically grown foods since the lowest level of the nutrients in the hydroponic FAVs is the highest level of the similar crops grown in the soil‐system. Even though it might take quite some time for farmers to master the operations that should be carried out in the hydroponic farms for better nutrient accumulation and to fill the gaps in the variation of nutrient levels in the same food crops obtained from different farms, hydroponically grown FAVs can adequately provide the nutrients in the recommended amounts for better health and nutritional outcomes of consumers as shown in this study. Considering that the hydroponic system can be set up using different techniques, this study gives evidence that the food crops that are grown in the NFT have a considerably high number of antioxidants compared to the crops grown in the soil‐based system. Scientifically, the results of this study show that the precise control of the environment in HFS could be the key to a greater nutrient accumulation in FAVs, which is beneficial to the health and nutrition of consumers, hence a reduction in the micronutrient deficiencies that count in the triple burden of malnutrition across the world.

This study proposes actions to be taken in the promotion of HFS and further research as follows:

### 5.1 Recommendations for Promotion of Hydroponic Farming

Since very few farmers in Kenya are using this system to grow crops because of unawareness and high startup costs, this study suggests that both commercial and smallholder farmers should be sensitized and trained about HFS so that they are equipped with the knowledge of setting up and managing crops in HFS. Additionally, the government should subsidize the materials required for setting up hydroponic farms and nutrients applied in the water to provide minerals for the proper growth of food crops.

### 5.2 Recommendations for Further Research

Since the level of antioxidants in the hydroponically grown spinach, tomatoes, and strawberries was higher than in the soil‐based samples, a study should be conducted to examine the nutrient level in other food crops so as to ascertain whether the accumulation is high across all the food crops grown in this system. Also, the nutrient levels in food crops grown in other hydroponic system techniques besides NFT should be determined to know whether they promote nutrient accumulation as in the technique examined in this study.

## Author Contributions

**Rhodah Nekesa:** conceptualization (lead), data curation (lead), formal analysis (lead), funding acquisition (lead), investigation (lead), methodology (equal), project administration (lead), resources (equal), software (equal), validation (equal), visualization (lead), writing – original draft (lead), writing – review and editing (equal). **Lucy G. Njue:** conceptualization (supporting), data curation (supporting), formal analysis (supporting), investigation (equal), methodology (supporting), project administration (supporting), resources (supporting), software (supporting), supervision (lead), validation (lead), visualization (equal), writing – original draft (supporting), writing – review and editing (lead). **George O. Abong:** conceptualization (supporting), data curation (supporting), formal analysis (supporting), investigation (supporting), methodology (supporting), project administration (supporting), resources (supporting), software (supporting), supervision (supporting), validation (supporting), visualization (supporting), writing – original draft (supporting), writing – review and editing (supporting).

## Ethics Statement

The authors have nothing to report.

## Consent

The authors have nothing to report.

## Conflicts of Interest

The authors declare no conflicts of interest.

## Data Availability

The data upon which the results of this study are based is available upon request.
